# Does written informed consent adequately inform surgical patients? A cross sectional study

**DOI:** 10.1186/s12910-018-0340-z

**Published:** 2019-01-07

**Authors:** Erminia Agozzino, Sharon Borrelli, Mariagrazia Cancellieri, Fabiola Michela Carfora, Teresa Di Lorenzo, Francesco Attena

**Affiliations:** Department of Experimental Medicine, Campania University L. Vanvitelli, Section of Hygiene Via L. Armanni 5, 80138 Naples, Italy

**Keywords:** Informed consent, Medical ethics, Surgery, Hospital, Italy

## Abstract

**Background:**

Informed consent (IC) is an essential step in helping patients be aware of consequences of their treatment decisions. With surgery, it is vitally important for patients to understand the risks and benefits of the procedure and decide accordingly. We explored whether a written IC form was provided to patients; whether they read and signed it; whether they communicated orally with the physician; whether these communications influenced patient decisions.

**Methods:**

Adult postsurgical patients in nine general hospitals of Italy’s Campania Region were interviewed via a structured questionnaire between the second and seventh day after the surgery at the end of the first surgical follow up visit. Physicians who were independent from the surgical team administered the questionnaire.

**Results:**

The written IC form was given to 84.5% of those interviewed. All recipients of the form signed it, either personally or through a delegate; however, 13.9% did not know/remember having done so; 51.8% said that they read it thoroughly. Of those who reported to have read it, 90.9% judged it to be clear. Of those receiving the written consent form, 52.0% had gotten it the day before the surgery at the earliest 41.1% received it some hours or immediately before the procedure. The written IC form was explained to 65.6% of the patients, and 93.9% of them received further oral information that deemed understandable. Most attention was given to the diagnosis and the type of surgical procedure, which was communicated respectively to 92.8 and 88.2% of the patients. Almost one in two patients believed that the information provided some emotional relief, while 23.2% experienced increased anxiety. Younger patients (age ≤ 60) and patients with higher levels of education were more likely to read the written IC form.

**Conclusions:**

The written IC form is not sufficient in assuring patients and making them fully aware of choices they made for their health; pre-operative information that was delivered orally better served the patients’ needs. To improve the quality of communication we suggest enhancing physicians’ communication skills and for them to use structured conversation to ensure that individuals are completely informed before undergoing their procedures.

**Electronic supplementary material:**

The online version of this article (10.1186/s12910-018-0340-z) contains supplementary material, which is available to authorized users.

## Background

Informed consent (IC) is a process by which a physician interacts with a patient, enabling the latter to make a knowledgeable decision regarding the treatment of his or her disease. IC consists not only of the form that patients must read and sign, it also involves oral communication that helps physicians establish a stronger relationship with the patients, which is considered by some to be a prerequisite for well-reasoned decision-making [1]. Moreover, two distinct but interrelated components characterize IC: the information about risks, benefits and alternatives and consent to undergo the proposed surgical procedure.

Italian jurisprudence has further broadened the meaning of IC to include information on supplies and equipment—as well as their service records—so that patients can opt to transfer to better-equipped facilities [2]. Despite these guidelines, implementing comprehensive IC is elusive.

Seeking informed consent is often a formal act in which a patient’s signature is obtained, with physicians believing that an important obligation has been fulfilled regardless of whether the patient has been provided with adequate information about the medical intervention that is about to take place [3].

Challenges and limitations of IC are widely discussed in different health care settings for different patients typologies [4–9]. The four principles of biomedical ethics (autonomy, beneficence, nonmaleficence and justice) are generally taken into account in these discussions [10]. Among them, the more pertinent to the ethics of IC is the principle of autonomy for which the person has the right, at all ages and stages of life, to have for himself, to the extent permitted by ethical evidence and by law, choosing whether to accept or refuse the ad, also offered from outside, on its health. And that it Is possible only as consequences of an adequate information.

We must remain mindful that there are patients who will not to be informed, who will not participate in treatment decisions and who will experience anxiety or other negative effects (this is known as “nocebo effect”), especially if they become aware of serious side effects due to surgery [11–14].

Surgical IC is particularly critical for the following reasons: the acute and the particularly vulnerable condition of patients; the urgency of the processes that must take place; the high technological level; the multiplicity of critical points of the process that can cause serious harm to the patient. Consequently, a greater difficulty arises for the patient to understand all these aspects and to decide accordingly, which includes consideration of any alternatives to the surgery [15, 16].

We explored whether the written IC form was delivered to patients, whether they signed the consent form and whether they read and understood the information about the surgical intervention. We also investigated verbal communication between patients and physicians and whether it affected patient decision making.

## Methods

### Setting

An epidemiological cross-sectional study was conducted between January 2016 to June 2016 to assess the quality of the IC process at nine general hospitals within Italy’s Campania Region. We selected these facilities to ensure representative coverage of the entire region territory as well as the representative participation of different general-hospital typologies. Four of the facilities are known as specialized hospitals (*aziende ospedaliere),* two are local hospitals (*presidi ospedalieri)*, one is a teaching hospital (*azienda ospedaliera universitaria)*, and two are private hospitals.

### Participants and Data Collection

Post surgery adult patients admitted in general surgery and giving the written consent to participate were included in the study. All patients were recruited in general surgery departments and interviewed via a structured questionnaire between the second and the seventh day after surgery, at the end of their first surgical follow up visit. Children under 18 years of age and patients who required intensive care or were taken back into surgery were excluded. Subjects were interviewed by one of four physicians adequately trained. All of them are specialized in Public Health. Epidemiology and Hospital Organization, and were independent from the surgical team and from the hospital. All the patients were interviewed in a room where their privacy could be ensured, and their answers remained confidential.

### Questionnaire

The questionnaire, which was divided into four sections, was formulated after an extensive literature search. The four questionnaire sections were as follows:

- Section 1: Descriptive characteristics of the study participants (*n* = 6 questions)
- Section 2: Information on the delivery, signing, reading and comprehensibility of the written IC form (*n* = 6 questions)
- Section 3: Additional information (acquired orally) on the explanation of the consent and on the effect of the written and oral information (*n* = 11 questions)
- Section 4: Information on the surgery outcome and on the post-surgical period (*n* = 4 questions)

### Sample Size

The sample size was estimated to be at least 400 subjects, assuming a 50% of expected prevalence of the most important variables (delivery, reading and understanding the IC form), with precision of 5% and level of significance of 95%.

### Data analysis

Descriptive statistics were computed using SPSS 21. The prevalence of delivery, reading and understanding of IC were calculated. Then, several bivariate analysis were performed to determine whether socio-demographics characteristics are in relationship with delivery, reading and understanding IC. Furthermore, we checked whether patients to whom the IC form had been explained or who had received additional oral information were more satisfied. Stratification analysis was used when crude Odds Ratio (OR) were statistically significant in bivariate analysis.

## Results

Among the 632 patients enrolled from the various hospitals, 72 (11.4%) did not adhere to the survey. Among the remaining 560 participants, 65.2% were female and 96.2% were Italian. Just over a third (65.5%) were under 60 years of age, 68.8% were married and only 12.0% had graduated; 85,4% of patients underwent a surgical procedure of medium or high complexity (Table 1).

**Table 1:** Socio-demographics characteristics of patients (*n* = 560)

	n	%
Sex		
Female	365	65.2
Male	195	34.8
Total	560	100.0
Age		
18–40	206	36.8
41–60	161	28.7
61–80	163	29.1
> 80	30	5.4
Total	560	100.0
Marital Status		
Married	385	68.8
Unmarried	104	18.6
Widow /Widower	54	9.6
Separate/Divorced	17	3.0
Total	560	100.0
Education		
Illiterate	5	0.9
Primary School	114	20.3
Middle School	188	33.6
High School	186	33.2
Degree	67	12.0
Total	560	100.0
Nationality		
Italian	539	96.2
Foreign	21	3.8
Total	560	100.0
Surgical Complexity		
Low	82	14.6
Middle	275	49.1
High	203	36.3
Total	560	100.0

Most respondents (84.5%) personally received a written IC form or they reported that they delegated to a parent or a relative, 1.6% declared that they did not get it, and the others (13.9%) did not recall receiving it. Among those patients who did receive the IC form, all signed it personally or through a relative or parent, but only 51.8% reported having read it thoroughly. Among those who read the IC, 90.9% judged it to be clear and 9.1% deemed it partially understandable. No one considered it incomprehensible. Approximately half of patients received the written IC form a day before surgery (at the earliest), while 41.1% received it within some hours or immediately before surgery. Forty-five percent of patients receiving the written forms were given them by the surgeon who performed the procedure (Table 2).

**Table 2:** Modalities of acquisition of written IC (*n* = 560)

	n	%
Have you received a written IC form?		
Yes	473	84.5
I don’t know / I don’t remember	78	13.9
No	9	1.6
Total	560	100.0
Who signed it?^a^		
Patient	448	94.7
Patient + relative	18	3.8
Relative	5	1.1
Parent	2	0.4
Total	473	100.0
Did you read it?^a^		
Yes	245	51.8
No, I did not want	167	35.3
No, due to lack of time	31	6.6
Partially / Distractedly	30	6.3
Total	473	100.0
Was it understandable?^b^		
Yes	249	90.9
Partially	25	9.1
No	0	0.0
Total	274	100.0
Time before the surgery^a^		
Immediately before	81	17.1
Some hours before	113	24.0
The day before	163	34.5
> 1 day	83	17.5
They can’t remember	33	6.9
Total	473	100.0
Who delivered it?^a^		
Operative surgeon	212	44.8
Other surgeon	160	33.8
Nurse	34	7.2
Anesthetist	13	2.8
Administration	2	0.4
I don’t remember	52	11.0
Total	473	100.0

^a^including only those who received it

^b^including only those who received and read it

Not all patients had the written consent for explained to them; it was explained orally only to 65.6%; however, 93.9% of patients received further oral information; among these 68.6% judged this information to be incomplete, and only 31.4% considered it complete.

The information was provided progressively during pre-operative examinations (66.7%) and, according to most (97.1%) recipients were understandable, as shown in Table 3.

**Table 3:** Explanation of the written IC and further oral information (n = 560)

	n	%
Was the consent explained upon delivery?		
Yes	367	65.6
No	59	10.5
Partially	31	5.5
No, I designated a relative	14	2.5
I have not signed / I don’t remember	89	15.9
Total	560	100.0
Did you receive further oral information besides the written consent?		
Yes	526	93.9
No	20	3.6
Given to a relative	14	2.5
Total	560	100.0
Degree of completeness of the oral information^a^		
Partial	361	68.6
Complete	165	31.4
Total	526	100.0
When was the further oral information provided?^a^		
Progressively during pre-surgery examination	351	66.7
On admission	94	17.9
On delivery of IC form	44	8.3
Before entering the operating room	33	6.3
At the first examination	3	0.6
The day after surgery	1	0.2
Total	526	100.0
Was it understandable?^a^		
Yes	511	97.1
Partially	15	2.9
No	0	0.0
Total	526	100.0

^a^among those who received information

Most attention was given to the diagnosis (Table 4), communicated to 92.8% of patients, and to the type of surgical procedure (88.2%); less attention was given to the prognosis (74.2%), post-operative progress (68.6%), benefits of the surgery (68.0%), consequences of a missed treatment (64.6%), existence of alternative therapeutic programs (63.2%), chances of success (61.8%), and possible surgical complications (53.6%).

**Table 4:** Type of oral information delivered by physician (*n* = 560)

	n	%
Diagnosis	520	92.8
Type of surgery	494	88.2
Prognosis	416	74.2
Post-operative progress	384	68.6
Benefits of surgery	381	68.0
Outcome of non treatment	362	64.6
Alternatives to the proposed surgery	354	63.2
Chances of success of the surgery	346	61.8
Potential complications of the surgery	300	53.6

Among patients who received both written consent and oral information, only 19.6% were “a lot” or “somewhat” influenced to undergo to the surgery, whereas 69.6% would have consented to the surgery with or without the additional information (Table 5).

**Table 5:** Effects of the written IC and the oral information on the patient’s decision making (*n* = 444)

	n	%
Written IC and oral information influenced the decision to proceed to the surgery?^a^		
A lot	41	9.2
Somewhat	46	10.4
A little	48	10.8
No	309	69.6
Total	444	100.0
Which one mainly influenced your decision?^a^		
Oral information	90	66.7
Both	28	20.7
I don’t know	14	10.4
Written IC form	3	2.2
Total	135	100.0
Compared to the information received. What would you want to know?^b^		
More	55	10.5
No more, no less	464	88.2
Less	7	1.3
Total	526	100.0
Have you had the opportunity to ask any questions?		
Yes, and I had exhaustive answers	361	64.4
Not asked questions	183	32.7
Yes, without satisfactory answers	16	2.9
Total	560	100.0
Consequences of the received information^b^		
Relief/Improvement in symptoms	262	49.8
Indifference	142	27.0
Anxiety/Worsening of the symptoms	122	23.2
Total	526	100.0

^a^including only those who received both written consent and oral information and only those whose decision had been influenced

^b^including only those who received further oral information

Among those who claim to have been influenced, 66.7% declared that they were mainly persuaded by the oral information, and 2.2% opined that they were more influenced by the written IC form. Among patients who received information, 88.2% were satisfied and did not believe they were in need of further details and, in fact, 64.4% of them reported that they had the opportunity to seek further information to answer their questions. Almost one in two patients (49.8%) were relieved to get the information, while 23.2% experienced an increase in anxiety because of the knowledge acquired.

Table 6 shows outcomes for patients in this study. Notably, 95.2% of respondents had no complications following surgery. Sixty-two percent said that their post-operative progress conformed to what was explained before the surgery, but almost a third (31.5%) reported that they did not receive information about post-operative progress. Nearly all patients (93.6%) were given instruction on self-care after discharge and, of those, 94.2% followed the guidance. Only 3.6% of the patients did not receive instructions, and 2.8% wanted to have received more information.

**Table 6:** Health outcome and coherence between indication and outcome after discharge (*n* = 560)

	n	%
Outcome of the surgery		
Successful	533	95.2
Complications	27	4.8
Total	560	100.0
Coherence of the information received with the real post-operative progress		
Coherent	349	62.3
Worse than expected	18	3.2
Better than expected	17	3.0
No information	176	31.5
Total	560	100.0
Instructions on the behaviors to keep after the discharge		
Received	524	93.6
Yes, but more Instructions needed	16	2.8
Not received	20	3.6
Total	560	100.0
Adherence to the post-operative care indications		
Yes	511	91.3
Partially	16	2.9
No	33	5.8
Total	560	100.0

Younger age (≤60) and a higher level of education (high school or degree) were the only socio-demographic characteristics statistically associated with reading of the IC form, also after stratification for age (< 60 and >  60) of education level with Mantel-Haenszel test (Table 7).

**Table 7:** Relationship between reading written IC with age and education level of patients and Mantel-Haenszel test (M.H)

		Yes/Total	Reading %
Age	> 60	50/155	32.2
	≤ 60	194/316	61.4
	Total	244/471	51.8
OR = 3.33 (CI 95%: 2.22–5.01) *p* = 0.000			
Education	< High School	102/247	41.3
	≥ High School	142/224	63.4
	Total	244/471	51.8
OR = 2.46 (CI: 1.70–3.57) *p* = 0.000			
Mantel-Haenszel test			
Age > 60	Education		
	< High School	30/112	26.8
	≥ High School	20/43	46.5
	Total	50/155	32.3
OR = 2.37 (CI 95%: 1.14–4.92) *p* = 0.001			
Age ≤ 60	Education		
	< High School	72/135	53.3
	≥ High School	122/181	67.4
	Total	194/316	61.4
OR = 1.81 (CI 95%: 1.14–2.86) *p* = 0.001			

M.H. test stratified for age OR = 1.94 (C.I.1.32–2.90) *p* = 0.001

Patients to whom the IC form had been explained or who had received additional oral information stated that they were more satisfied compared to those who had only received the written IC, *p* = 0,01 (Table 8). We have considered as “satisfied” those patients who answered “no more no less” to the question “Compared to the information received, how much would you want to know?” and “not satisfied” those patients who answered “more” to the same question. Patients who responded that they wanted to have less information (7 subjects) were excluded.

**Table 8:** Relationship between patients satisfaction (not satisfied/satisfied) and oral explanation of IC form

Oral explanation of ICform	Not satisfied	Satisfied	Total	O.R.	C.I. 95%	*p*
	n / %	n / %				
Not explained	25 / 15.6	135 / 84.4	160	**1,00**		
Explained	30 / 8.4	329 / 91.6	359	**2,03**	1,15-3,58	0,01
Total	55 / 10.6	464 / 89.4	519			

## Discussion

Complete information before an invasive procedure is an ethical requirement, and it is very important to involve the patient in decision-making regarding the treatment. Well-informed patients are generally more satisfied and file fewer legal claims [17–21]. Conversely, patients who were not informed about the risks of surgery regretted the decision after the surgery [11, 22–24].

We analyzed the four elements contributing to correct utilization of written IC forms: delivery, signature, reading and comprehensibility. Delivery and signature are formal and compulsory actions that comply with Italian laws. Reading and comprehensibility are key actions that allow the patient to become aware of the risk benefit of the practical intervention. Therefore, in our study the main shortcoming of the written IC process has been that almost all patients received and signed it, but only half of them read it adequately.

Possible reasons for the findings are: first, many patients showed scarce interest in the IC document, probably preferring to rely on the surgeon’s expertise or they would not be able to understand. In our study, the patients with a higher educational level and who were not older than 60 years old were more likely to read the IC form [25]. Secondly, surgeons might have shown a lack of interest in the document, sought signatures without giving adequate support and motivation to the patients and hence did not explain to them the importance of reading the document [22, 26–28]. Thirdly, almost half the patients received the form immediately or just a few hours before the surgical procedure—when they were more stressed and vulnerable, with little time to read and reflect on it [13]. A probable reason for this is that in the Italian culture, physicians regard the delivery and the signing of written IC form as a fulfillment of the law [3], while more importance is given to oral information. Indeed, two-thirds of patients reported that at the handover, the consent was also explained, and almost all received information beyond what was in the IC form at various times before the surgical intervention.

In agreement with findings of other authors [29, 30], when delivering oral information, patients stated that the surgeons focus principally on the diagnosis and on the type of surgical procedure; a lower percentage of patients reported being informed about other aspects of the treatment, such as prognosis, consequences of a missed treatment or the possible surgical complications. No information was provided about possible deficiencies in the facilities’ biomedical equipment or specific diagnostic tools that can reduce safety aspects of the procedures [3]. Therefore to improve the quality of communication with the written IC, we agree with Ghulam, who suggests that a structured conversation helps physicians establish relationships with the patients, facilitates the documentation and offers a valid legal proof for patients and physicians regarding the adequacy of the information provided [13].

Though there is an ethical imperative to inform the patient, it is not always reassuring to the patient: indeed, about one fourth of patients experienced nocebo effects, such as increased anxiety because of the acquired knowledge [11–14]. Moreover, the majority of patients were hardly influenced or not at all influenced by the written and oral information, having previously decided to undergo the surgery. These findings recall a well-known dilemma of IC. If IC is too detailed, it might violate the principle of nonmaleficence by causing nocebo effect. If it is less detailed, it might violate the principle of autonomy not allowing a conscious choice by the patient. Wells and Kaptchuk [14] try to overcome this dilemma by proposing a *contextualized informed consent*. They consider it an ethical procedure whereby a provider takes into account the possible side effects, the typology of patient, and the diagnosis involved, to provide information tailored in view to reduce expectancy-induced side effects, and, at the same time, to respect patient autonomy and conscious choice. Therefore, the previous relative definition of “complete” IC, as reported at the beginning of the discussion, should be concluded with the statement “considering the possibility of inducing nocebo effect”.

The main limitation of the present survey is the potential recall bias because the information was obtained through face-to-face interview many days after the delivery of the written IC and after discharge that caused many answers “I don’t know/ I don’t remember” reported in the tables. The patients satisfaction has not been collected as direct question, but has been evaluated as surrogate variable, interpreting the answer to a different question.

Furthermore, patients who had required intensive care or had been brought back into surgery were excluded. Therefore, the results would not be generalized to patients in more severe settings.

## Conclusion

Written IC is essential in medicine in order to ensure that patients have the needed information to make an aware choice and consenting to treatment. Furthermore, various authors have demonstrated that written information is best understood by patients who can read the information many times and can discuss it with relatives and friends [3, 13], who are reassuring points of reference for post-operative progress. Oral information is not always easily absorbed because of the escalated stress level just prior to surgery [13].

In our experience, written information has not provided patients with adequate decision-making tools for imminent health matters, while pre-operative oral information was better suited to meet patients’ needs.

Finally, we agree with authors who think it is necessary that physicians also enhance their communication skills to enable them to build alliances with patients in order to become effective partners and ensure that patients can make informed choices in accordance with their own feelings and values [3, 22, 31, 32]. Because of this, we must implement communication training for doctors as an integral part of clinical skills to reach a complete patient compliance. This must be incorporated into training at all levels, including postgraduate medical programs, in order to improve physicians’ listening skills and develop interactive communication skills. Such a trained physician can help patients in defining what is the best choice to make in order to improve their own health status, in accordance to their experiences and values and within a relationship of trust and collaboration (shared decision making) [33]. This seems to be the main way to ensure patients’ “self-determination” in health care.

## Additional files

Additional file 1: Questionnaire: Patient’s informed consent procedure. (PDF 497 kb)

Additional file 2: Consent Data base. (XLS 164 kb)

## Abbreviation

IC: Informed Consent
M.H: Mantel-Haenszel test
OR: Odds Ratio
