# Supplementary material for: Does written informed consent adequately inform surgical patients? A cross sectional study
Source: BMC Med Ethics. 2019 Jan 7;20:1. doi: 10.1186/s12910-018-0340-z (PMC6323683; doi:10.1186/s12910-018-0340-z)
Supplement: Supplementary file 1 — Questionnaire: Patient’s informed consent procedure. (PDF 497 kb) [file 12910_2018_340_MOESM1_ESM.pdf]

Patient's informed consent to surgical procedure

Date of the interview________ Hospital______________________
Department____________________

1) **Sex**

☐ M ☐ F

2) **Age**

☐<18☐18-40☐41-60☐61-80☐>80

3) **Nationality**

☐Italian
☐Other_______________

4) **What is your marital status?**

☐Unmarried☐Married
☐Widow /Widower☐Separate/Divorced

5) **What is your higher level of education?**

☐Primary School☐Middle School (professional qualifications)
☐High School☐College/University

6) **Date of surgical procedure_________________**

7) **Type of admission_________________**

☐Ordinary admission
☐Day Hospital

WRITTEN INFORMATION

8) **Have you received a written Informed Consent (IC) form?**

☐Yes
☐No

9) **How long before the surgical procedure have you received the IC form?**

☐**Just before**

☐**Some hours before**☐**The day before**☐**Other**____

10) **Who delivered the IC form?**

☐**Operative surgeon**

☐**Head physician**

☐**Other physician of the surgical unit**

☐**Other**_____________________________

11) **Who signed the IC form?**

☐**Patient**

☐**Parent (if minor)**

☐**Legal representative (if interdicted or unable person)**

☐**Other**___________

12) **Did you read the IC form before signing it?**

☐**Yes**

☐**No (They just made me sign it; due to lack of time)**

☐**No (I did not want to read it)**

☐**Partially. Why?**

____

☐**Distractedly. Why?**

____

13) **Was the IC form understandable?**

☐**Yes**

☐**No**

☐**Partially**

☐**Other/Notes**____

ORAL INFORMATION

1) **Was the IC form explained during a conversation?**

☐**Yes**

☐**No**

☐**Partially**

☐**Notes**____

2) **Did you receive further oral information about:**

☐**Your condition: diagnosis?**
☐**Prognosis?**
☐**Type of the proposed treatment?**
☐**Expected post-operative progress and possible recovery issues?**
☐**Potential benefits of the proposed treatment?**
☐**Potential inconveniences (complications)of the proposed treatment?**
☐**Alternatives to the proposed treatment?**
☐**Chances of success of the proposed treatment?**
☐**Possible outcomes of a missed treatment?**

3) **When did you receive the further oral information?**

☐**Progressively during pre-operative examination**
☐**On admission**
☐**Before entering the operating room**
☐**On delivery of IC form**
☐**Other______________**

4) **Did you understand what was proposed to you, was the language understandable?**

☐**Yes**
☐**No**
☐**Partially_______________________________________**
☐**Notes_______________________________________**

5) **Did IC form and/or oral information influence the decision to proceed to the surgical procedure?**

☐**A lot**☐**Somewhat**☐**A little**☐**No**

6) **If yes, which one mainly influenced your decision?**

☐**Written IC form**
☐**Oral information**
☐**Both**

7) **Compared to the information received, how much would you want to know?**

☐**More**
☐**No more, no less**
☐**Less**

☐**Less about risks and complications**

8) **Have you had the opportunity to ask any questions and have you obtained exhaustive answers?**

☐**Yes, I asked questions and I had exhaustive answers**
☐**Yes, I asked questions but I didn't have exhaustive answers**
☐**No, I didn't ask any questions**

9) **Received information caused you**

☐**More anxiety–your health**
☐**Worsening of the symptoms**
☐**More relief regard your health**
☐**Improvement in symptoms**

HEALTH OUTCOMES

10) **What was the outcome of your surgical procedure?**

☐ **Successful/normal**
☐ **Complications____**
☐ **Hospital-acquired infections**

11) **Were the information received before the surgical procedureabout the post-operative progress correct?**

☐**Yes**
☐**No, progress worse than expected**
☐**No, progress better than expected**

12) **Did you receive instructions on the behaviors to keep after the discharge?**

☐**Yes**
☐**Yes, but I wanted more information**
☐**No**

13) **Did you follow post-operative care indications?**

☐**Yes**
☐**No**
☐**Partially____**
